# Placenta accreta: étude descriptive rétrospective de 46 cas pris en charge au Service de Gynécologie-obstétrique du Centre Hospitalier Universitaire Farhat Hached de Sousse, Tunisie

**DOI:** 10.11604/pamj.2024.47.147.38111

**Published:** 2024-03-27

**Authors:** Badra Bannour, Imen Bannour, Sarra Hachicha, Maroi Bannour, Hedi Khairi

**Affiliations:** 1 University of Sousse, Faculty of Medicine of Sousse, Obstetrics Gynecology Department of the Farhat Hached University Hospital Center of Sousse, Tunisia,; 2 “LR12ES03” Research Laboratory, 4002 Sousse, Tunisia,; 3 Anesthesia Department, University Hospital Farhat Hached, Sousse, Tunisia

**Keywords:** Placenta accreta, césarienne, hémorragie du post-partum, pronostic maternel, Placenta accreta, cesarean section, postpartum hemorrhage, maternal prognosis

## Abstract

Le placenta accreta est une anomalie d'insertion placentaire rare mais grave. Ce travail a pour objectif d'analyser les caractéristiques épidémiologiques, cliniques, paracliniques et évolutives du placenta accreta, d'étudier la prise en charge thérapeutique et d´évaluer la morbidité et la mortalité maternelle et néonatale. Nous avons mené une étude rétrospective, descriptive, au service de gynécologie obstétrique du CHU Farhat Hached de Sousse regroupant les cas de placentas accretas confirmés histologiquement sur une période de 4 ans allant du 1^er^ janvier 2015 au 31 décembre 2019. Un canevas d'étude a été établi et dans lequel les données épidémiologiques, cliniques, paracliniques, thérapeutiques et évolutives ont été relevées à partir des dossiers des malades et des comptes-rendus opératoires. Dans notre série, nous avons recensé 46 cas du placenta accreta. L´âge moyen de nos patientes était de 35± 4,61 ans. Chacune de nos patientes avait un utérus cicatriciel. Le terme moyen d'accouchement était 34 semaines d'aménorrhées. Le mode d'accouchement était une césarienne pour toutes nos patientes. Une hystérectomie de première intention a été réalisée chez 40 patientes et un traitement conservateur chez 6 patientes. Seize patientes ont développé des complications maternelles. Aucun cas de décès maternel n'a été observé. Le placenta accreta est une pathologie rare associée à une morbidité materno-fœtale importante.

## Abstract (en)

Placenta accreta is a rare but serious placental attachment abnormality. The aim of this study is to analyze the epidemiological, clinical, para-clinical and evolutionary features of placenta accreta, to investigate the therapeutic management and to assess maternal and neonatal morbidity and mortality. We conducted a retrospective, descriptive study of patients with histologically confirmed placenta accreta in the obstetrics and gynaecology department of the Farhat Hached University Hospital in Sousse, over a 4-year period from 1^st^ January 2015 to 31^st^ December 2019. The epidemiological, clinical, paraclinical, therapeutic and evolutionary data were collected from patients´ medical records and operative reports. In our series, we identified 46 cases of placenta accreta. The average age of our patients was 35±4.61 years. Each of our patients had a scarred uterus. The average term of delivery was 34 weeks of amenorrhoea and the mode of delivery was caesarean section for all our patients. First-line hysterectomy was performed in 40 patients and conservative treatment in 6. Sixteen patients developed maternal complications. No maternal death was observed. Placenta accreta is a rare condition associated with significant maternal and foetal morbidity.

## Introduction

Le placenta accreta est une anomalie d'insertion placentaire rare mais grave. Il résulte d'une absence de la caduque basale responsable alors d'un envahissement anormal du myomètre par les villosités choriales [1]. Selon le degré histologique de l'invasion myométriale, on définit trois grades. L'envahissement de la surface du myomètre par les villosités choriales définit le placenta accreta. Dans le placenta increta, les villosités choriales s'étendent à tout le myomètre jusqu´au séreuse sans la dépasser. Enfin, le placenta percreta est rencontré quand les villosités choriales dépassaient le myomètre pour envahir la séreuse utérine voire les organes de voisinage comme la vessie [2]. Le placenta accreta est associé à une morbidité et une mortalité maternelle importante [3-5].

Le traitement de première intention a longtemps été radical avec la réalisation d´une hystérectomie. L'objectif de ce traitement radical était de limiter le risque hémorragique pour réduire la morbidité maternelle. La perte de la fertilité reste l´inconvénient majeur du traitement radical [6].

Grace aux progrès des techniques hémostatiques tant médicamenteuses que chirurgicales, une alternative conservatrice en laissant le placenta in situ est actuellement possible. Ce traitement conservateur permet de préserver la fertilité ultérieure et probablement de réduire les pertes sanguines et la morbidité de l'hystérectomie [6]. Cependant, cette approche ne doit être envisagée que chez les patientes hémodynamiquement stables qui souhaitent fortement préserver la fertilité et qui comprennent et acceptent les risques d´hémorragie retardée et d'infection [1,7]. L'objectif de notre étude est de décrire les caractéristiques épidémiologiques et les stratégies thérapeutiques du spectre du placenta accreta.

## Méthodes

**Type et cadre de l'étude:** il s'agit d'une étude rétrospective, descriptive, menée au service de gynécologie et obstétrique du CHU de Farhat Hached de Sousse sur une période de 4 ans allant du 1^er^ janvier 2015 au 31 décembre 2019.

**Participants à l'étude:** nous avons inclus toute patiente ayant accouché au CHU Farhat Hached de Sousse et chez qui nous avons diagnostiqué histologiquement un placenta accreta.

Les critères de non inclusion étaient les patientes qui ont subi une hystérectomie pour une suspicion de placenta accreta dans une autre maternité et qui ont été transférées au service de gynécologie obstétrique du CHU Farhat Hached de Sousse en post opératoire.

**Les sources de données:** les dossiers médicaux ont été examinés rétrospectivement.

**Les variables quantitatives:** les différentes variables recueillies et analysées pour chaque patiente étaient: l'âge maternel, les antécédents médicaux, chirurgicaux et gynéco-obstétricaux, l´examen clinique, les données de l´imagerie médicale, le type de chirurgie pratiquée, le nombre d´unités et le type de composants sanguins (CGR, PFC ou plaquettes) administrés en per opératoire et 48 heures après l´opération et les suites opératoires.

**Les méthodes statistiques:** les méthodes statistiques standards ont été utilisées pour le calcul des moyennes et des fréquences. La saisie et l´analyse des données ont été effectuées par le logiciel EPI info version 2020.

**Considérations éthique:** notre étude a été approuvée par le comité éthique.

## Résultats

### Caractéristiques générales de la population étudiée

Un total de 46 cas histologiquement prouvés a été inclus. L'âge moyen de nos patientes était de 35,11 ± 4,6 ans avec des extrêmes allant de 26 à 43 ans. Chacune de nos patientes avait un utérus cicatriciel. Aucun antécédent de placenta accreta n´a été noté (Tableau 1).

**Tableau 1:** description de la population étudiée

		Effectif	Pourcentage (%)
**Age maternel**	<30	5	10,8
	30-35	15	32,6
	>35	26	56,5
**Gestité**	2	2	4,3
	3	9	19,5
	4	17	36,9
	5	12	26
	6	6	13
**Parité**	1	4	8,6
	2	23	50
	3	12	26
	4	6	13
	5	1	2,1
**Antécédent de césarienne**	1	9	19.5
	2	22	47,8
	3	10	21,7
	4	4	8,7
	5	1	2,1
**Antécédent de curetage**	0	37	80,4
	1	6	13
	2	3	6,5
**Antécédent myomectomie**	0	0	0
**Antécédent de placenta prævia**	0	44	95,7
	1	2	4,3
**Type de grossesse**	Grossesse mono-fœtale	45	97,8
	Grossesse gémellaire	1	2,2

### Présentation clinique

**Figure 1:**
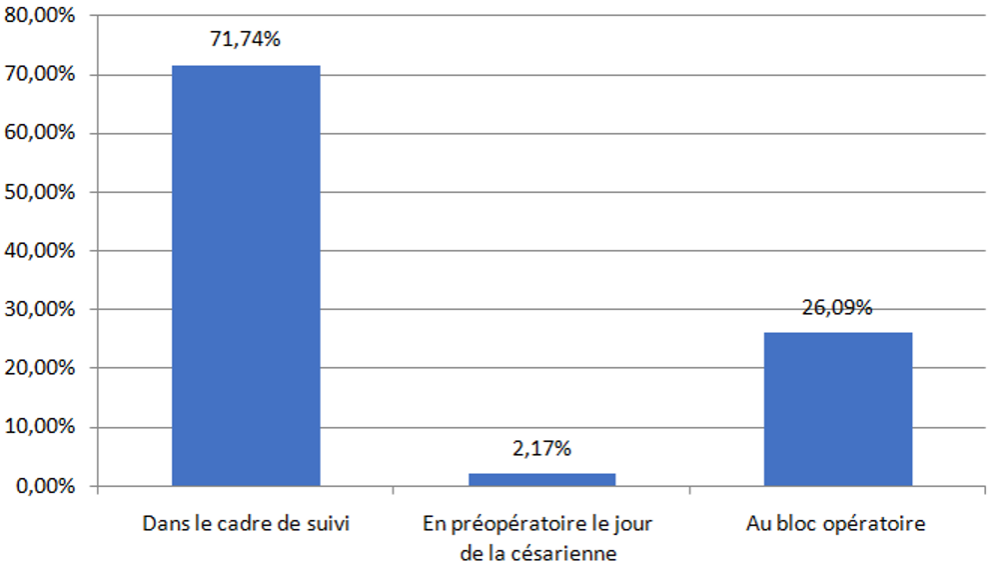
répartition selon les circonstances du diagnostic du placenta accreta

**Figure 2:**
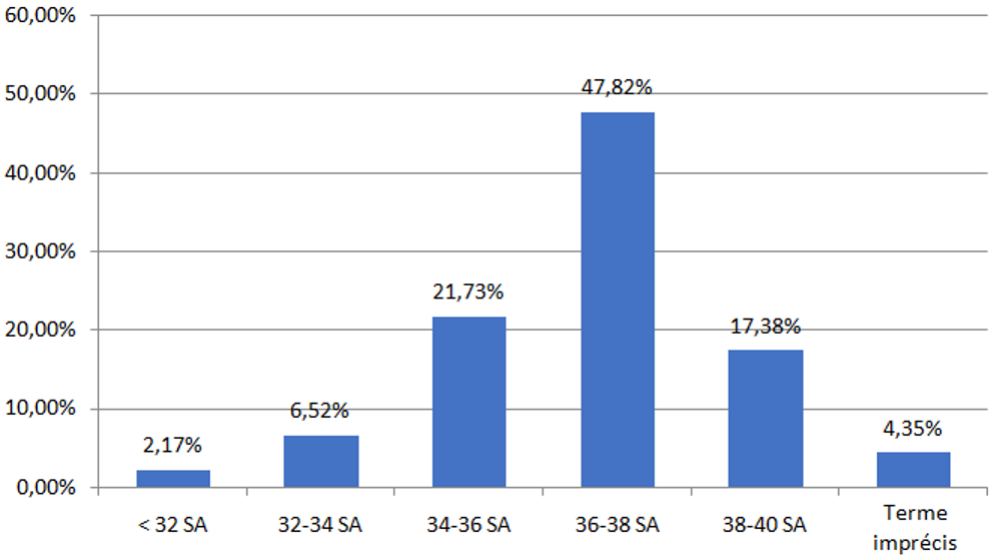
terme d'accouchement

Le diagnostic de placenta accreta a été fait en anténatal pendant le suivi de la grossesse dans 71,7% des cas (n=33). Le diagnostic a été fait en urgence le jour de la césarienne dans 28,2% des cas (n=13). Un peu plus de quarante-sept pourcent (47,2%) des patientes (n=22) étaient asymptomatiques jusqu´à l´accouchement. Une métrorragie du 3^e^ trimestre de faible abondance a été rapportée dans 36,9% des cas (n=17) et la survenue des contractions utérine a été rapportée dans 6,5 % des cas (n=3) (Figure 1). Le terme médian de la grossesse le jour du diagnostic du placenta accreta était de 34 SA avec des extrêmes allant de 22 SA à 40 SA. Concernant les données échographiques, le placenta était non prævia dans 10,8% des cas (n=5). Les signes d´accrétisation échographiques ont été retrouvés chez 67,4% de la population étudiée (n=31) et ont été absents chez 32,6% (n=15) (Tableau 2). La médiane du terme d´accouchement était de 37 SA avec des extrêmes allant de 27 SA+ 3 j à 40 SA (Figure 2).

**Tableau 2:** répartition selon les signes échographiques d’accréditation

		Effectif	Pourcentage (%)
**Type d'insertion placentaire**	Placenta non prævia	5	10,8
	Placenta prævia type I antérieur	2	4,3
	Placenta prævia type II antérieur	1	2,1
	Placenta praevia type III antérieur	6	13
	Placenta praevia type IV antérieur	27	58,6
	Placenta praevia type IV postérieur	5	10,8%
**Les signes échographiques d'accrétisation**	Disparition du liséré de sécurité entre placenta et myomètre	22	47,8
	Anomalie de l'interface utérus / mur vésical	9	19,5
	Lacunes intra placentaires vascularisées	30	65,2
	Vaisseaux perpendiculaires à la zone de suspicion d'insertion accreta se dirigeant vers la séreuse utérine	8	17,3
	Hyper vascularisation au niveau de l'interface utérus/vessie	23	50

### Prise en charge et résultats

**Figure 3:**
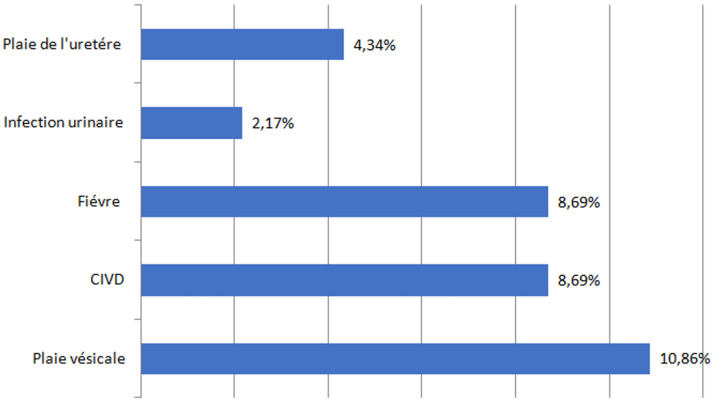
répartition selon les complications maternelles

Toutes les patientes ont accouché par césarienne. La césarienne a été programmée dans 58,7% des cas (n= 27), faite en urgence dans 41,3% des cas (n=19). Le traitement chirurgical était radical, en pratiquant d'emblée une hystérectomie d´hémostase, dans 40 cas (86,9%). Il a été associé à une ligature hypogastrique seule dans 60% (n=24), un packing seul dans 15% des cas (n=6) et l'association de ces deux techniques dans 25% des cas(n=10). Les résultats anatomopathologiques montraient qu'il s´agissait de 30 cas de placenta accréta (65,2%), 6 cas de placenta increta (13%), 4 cas de placenta percreta (8,7%). Le traitement était conservateur dans 6 cas (13%). Il a été fait en laissant en place une partie du placenta dans 5 cas (83,3%) et en laissant tout le placenta dans un seul cas (16,6%) avec une reprise chirurgicale et une hystérectomie programmée après 3 semaines faite sans incidents. Un traitement adjuvant au traitement conservateur a été indiqué dans 5 cas, réparti comme suit: une ligature des artères hypogastriques dans 2 cas (40%), un capitonnage seul dans 1 cas (20%), une triple ligature seule dans un seul cas (20%) et une association d´un capitonnage et une triple ligature dans un seul cas (20%). Vingt-huit (28) patientes subissant un traitement radical et 5 patientes subissant un traitement conservateur ont été transfusées (Tableau 3). Parmi nos patientes, 16 cas (34,8%), ont développé des complications maternelles (Figure 3). Le score d´Apgar moyen à 10 minutes a été de 8,33 ± 3,21 avec des extrêmes allant de 0 à 10.

**Tableau 3:** répartition des besoins transfusionnels selon le type anatomopathologique du placenta

	Accreta (n=30)	Increta (n=6)	Percreta (n=4)
**Moyenne d’Hb préopératoire (g/dl)**	11,3	10,6	12,2
**Nombre moyen de CGR**	2,7	4	2,5
**Nombre moyen de poches de PFC**	5,6	4	0
**Nombre moyen de culots plaquettaire**	7	0	0

## Discussion

En analysant les facteurs de risque de placenta accreta, un antécédent d'accouchement par césarienne était le facteur de risque le plus important. Toutes les patientes ont accouché par césarienne. La césarienne a été programmée dans 58,7% des cas (n= 27), faite en urgence dans 41,3% des cas (n=19). Le traitement chirurgical était radical, en pratiquant d'emblée une hystérectomie d'hémostase, dans 40 cas (86,9%). Parmi nos patientes, 16 cas (34,8%), ont développé des complications maternelles.

Le spectre d'adhésion placentaire est une affection grave due à une insertion anormale du placenta dans la paroi utérine. Son incidence ne cesse d'augmenter. Concernant les facteurs de risque, la césarienne est le premier facteur de risque en termes de fréquence. En fait, plusieurs auteurs ont conclu qu'il existe une relation proportionnelle entre le nombre de césariennes et le risque de placenta accreta [6]. L'association de cicatrices utérines et de placenta praevia augmente fortement le risque d'accrétisation [7]. Dans notre série, 89% des patientes avaient un placenta praevia.

L´échographie obstétricale est le test de dépistage prénatal le plus important [2,8]. Si l'échographie reste non concluante, une imagerie par résonance magnétique placentaire peut être envisagée. Le diagnostic de certitude reste anatomopathologique sur pièce d'hystérectomie [9]. Le Collège Royal de Gynécologie Obstétrique ainsi que le Collège Américain de Gynécologie Obstétrique recommandent une prise en charge multidisciplinaire de placenta accreta. Ainsi, devant une suspicion d´une invasion placentaire, un transfert pour un accouchement dans un établissement adapté est recommandé. Le Collège Royal de Gynécologie Obstétrique recommande un accouchement entre 35SA et 36SA. Le collège américain de gynécologie obstétrique recommande un accouchement à 34SA [10]. Dans notre série, le terme moyen d´accouchement était de 37SA. La césarienne - hystérectomie est considérée comme le gold standard de la prise en charge de l´invasion placentaire. L'objectif de ce traitement est de limiter le risque hémorragique pour réduire la morbidité maternelle. Cependant, la perte de la fertilité reste son inconvénient majeur [10]. Ce traitement radical n'est pas dépourvu de complications. Les plus fréquentes sont l'hémorragie et les lésions vésicales et urétrales. Ainsi, Eller *et al*. rapportaient un taux de transfusion (≥ 4 culots globulaires) de 42%, de cystotomie de 29%, de plaies urétérales de 7% et de complications infectieuses de 33% [11]. Une alternative conservatrice en laissant le placenta in situ est actuellement possible. Cette approche ne doit être envisagée que chez les patientes hémodynamiquement stables qui souhaitent fortement préserver la fertilité. La ligature vasculaire et l'embolisation par radiologie interventionnelle sont des alternatives supplémentaires au traitement conservateur [12].

Les limites de notre étude sont celles de toute étude rétrospective. Nous avons utilisé la confirmation histologique comme référence pour le diagnostic du placenta accreta. Les autres limites sont la brièveté de la période étudiée et l´absence de données sur un suivi à long terme.

## Conclusion

Dans notre étude, l´antécédent de césarienne était le facteur de risque commun pour toutes nos parturientes. La césarienne - hystérectomie est le traitement chirurgical le plus fréquent. Ce traitement radical n'est pas dépourvu de complications. Les plus fréquentes sont l´hémorragie et les lésions vésicales et urétrales. La thérapie transfusionnelle est une composante essentielle dans la prise en charge. Notre étude souligne l´importance d´avoir un centre national de référence de placenta accréta afin de garantir la meilleure prise en charge par une équipe multidisciplinaire bien entrainée sur cette pathologie.

### Etat des connaissances sur le sujet

- *Le placenta accreta présente une des causes d'hémorragie sévère de la délivrance;*
- *Le plus grand risque survient à l´accouchement;*
- *Si le placenta accreta n'est pas diagnostiqué, on peut assister à une hémorragie maternelle potentiellement catastrophique*.

### Contribution de notre étude à la connaissance

- *Le traitement chirurgical de première intention du placenta accreta est la cesarienne-hysterectomie;*
- *Ce traitement chirurgical n’est pas dépourvu de complications; les plus fréquentes sont l’hémorrhagie et les lésions vésicales et urétrales;*
- *La mortalité est faible si la prise en charge du placenta accreta se fait dans un centre de référence en Tunisie*.
